# Complete chloroplast genome sequence of the *Amygdalus Nana*

**DOI:** 10.1080/23802359.2019.1663768

**Published:** 2019-09-10

**Authors:** Yizhong Duan, Zhongyu Du

**Affiliations:** a Shaanxi Key Laboratory of Ecological Restoration in Northern Shaanxi Mining Area, Yulin University, Yulin, China;; b College of Chemistry & Materials Science, Northwest University, Xi’an, China;; c Breeding Base for State Key Laboratory of Land Degradation and Ecological Restoration in Northwest China, Ningxia University, Yinchuan, China;; d Ministry of Education Key Laboratory for Restoration and Reconstruction of Degraded Ecosystems in Northwest China, Ningxia University, Yinchuan, China

**Keywords:** *Amygdalus nana*, complete chloroplast genome, phylogenetics

## Abstract

Amygdalus nana is the research materials, and we used the Illumina HiSeq X Ten system to do sequencing, and used the complete chloroplast genomes of 12 species to constructed thephylogenetic tree. The results showed that the complete chloroplast genome of the *A. nana* was 158,596 bp in length, containing a largen single copy (LSC) region of 86,608 bp, a small single copy (SSC) region of 18,998 bp, and a pair of inverted repeats (IRs) region of 26,411 bp. The genome has a GC content of 36.7%. The LSC, SSC, and IR regions represent 54.61, 11.98, and 33.31% of the *A. nana* chloroplast genome length respectively. We annotated 130 genes, including 85 protein coding genes, 8 rRNA genes, and 37 tRNA genes. And the phylogenetic analysis suggested that the *A. nana* is closely related to *A. mongolica*.

*Amygdalus nana* (Rosaceae) is also called *Amygdalus Ledebouriana* Schleche, in China, *A. nana* is restricted to the northwest Xinjiang Uygur Autonomous Region (Tahan et al. 2009). The species is not only resistant to drought and cold, has very strong adaptability, but also it can be used as the breed of original material. In the early spring, it is a beautiful ornamental shrub (Lu and Bartholomew 2003). However, in recent years, due to the global climate changes and human activities, the existing of *A. nana* is declining. Although many areas have successfully introduced trials (Mei et al. 2014), there are many resources that we don’t know it can be used, such as the difference population of A. nana. and the difference analysis of adaptive evolutin,etc. At present, the research on *A. nana* is mainly concentrated on biological characteristics (Luo et al. 2009; Wang et al. 2010; Han et al. 2013), the research on the *A. nana* complete chloroplast genome (cp DNA) has not been reported. In this study, we choose the fresh leaves of the *A. nana*, analyzing the complete chloroplast genome with high-throughput sequencing technology. We hope to provide some help for the study of the *A. nana* in the future.

In September 2018, the fresh *A. nana* leaves were collected in Minqin Desert Botanical Garden, Gansu, China (103° 50′ E, 38° 38′ N; Height: 1378 m above sea level). The specimens of *A. nana* (Accession Number: 20180903Yl01) were deposited at the Herbarium of college of life science, Yulin University, Shaanxi province, China. The chloroplast genomic DNA was extracted from the fresh leaves according to a modified CTAB method (Doyle and Doyle 1987), we used the Illumina HiSeq X Ten system to do high-throughput sequencing, and the *Prunus pedunculata* complete chloroplast genome (MG869261) was used as the reference sequence to annotate. The *A. nana* complete chloroplast genome was annotated with the Geneious (Kearse et al. 2012). The physical map of the complete chloroplast genome was generated using OGDRAW (Lohse et al. 2013). The complete chloroplast genome sequences were aligned using MAFFT (Kazutaka et al. 2002), We used the MEGA v7.0 (Kumar et al. 2016) to construct a phylogenetic tree according to the neighbor-joining method, with a bootstrap value of 1000. At last, the annotated *A. nana* complete chloroplast genome sequence has been deposited into the GenBank database, and the accession number is MK764428.

The results showed that the *A. nana* complete chloroplast genome involves 158,596 bp, containing a largen single copy (LSC) region of 86,608 bp, a small single copy (SSC) region of 18,998 bp, and a pair of inverted repeats (IRs) region of 26,411 bp. The genome has a GC content of 36.7%. The LSC, SSC, and IR regions represent 54.61, 11.98, and 33.31% of the *A. nana* complete chloroplast genome length. We annotated 130 genes, including 85 protein coding genes, 8 rRNA genes, and 37 tRNA genes.

We used the complete chloroplast genomes of 12 species to constructed the phylogenetic tree, and the *Actinidia eriantha* (NC034914) and the *Actinidia arguta* (MF521827) complete chloroplast genome as outgroups (Figure 1). We aligned all 12 sequences using MAFFT. The results showed that the *A. nana* is closely related to *A. mongolica*.

**Figure 1.**
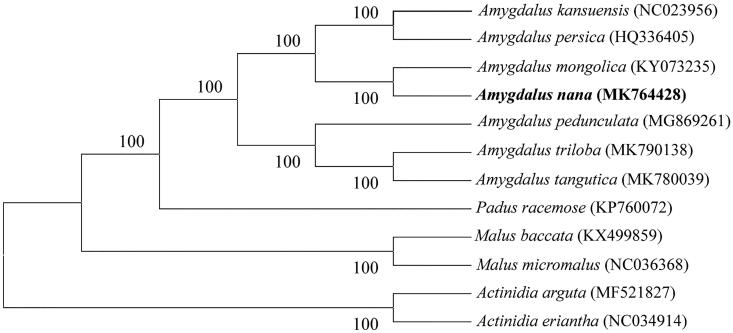
Phylogenetic tree constructed based on 12 species of chloroplast genomes. Accession numbers: *Malus baccata* (KX499859); *Malus micromalus* (NC036368); *Actinidia eriantha* (NC034914); *Actinidia arguta* (MF521827); *Padus racemose* (KP760072); *Amygdalus persica* (HQ336405); *Amygdalus pedunculata* (MG869261); *Amygdalus mongolica* (KY073235); *Amygdalus kansuensis* (NC023956); *Amygdalus triloba* (MK790138); *Amygdalus tangutica* (MK780039); *Amygdalus nana* (MK764428).
